# How does wheat straw‐derived biochar influence the nutrient pool of a site‐specific Luvisols in a laboratory incubation?

**DOI:** 10.1002/jeq2.70121

**Published:** 2025-12-11

**Authors:** Syazwan Sulaiman, Guillermo Hernandez‐Ramirez, Namasivayam Navaranjan, Zohrah Sulaiman

**Affiliations:** ^1^ Faculty of Agricultural, Life and Environmental Sciences University of Alberta Edmonton Alberta Canada; ^2^ School of Applied Sciences and Mathematics Universiti Teknologi Brunei Bandar Seri Begawan Brunei Darussalam

## Abstract

The impact of biochar on soil nutrient pool has been well‐studied in degraded and acidic soils, yet its effects in fertile soils such as Luvisols remain underexplored. To address this, two laboratory incubation experiments were conducted using biochar derived from wheat straw (*Triticum aestivum*)—Experiment 1 evaluated biochar produced at three pyrolysis temperatures (350°C, 500°C, and 650°C) with two residence times (1 and 2 h), whilst Experiment 2 examined the feasibility of different application rates (5 or 10 Mg ha^−1^) and placements (thorough mixing or surface broadcast). Biochar significantly increased exchangeable Ca, K, Mg, and Na concentrations compared to both control and straw‐amended soils, particularly with the higher temperature biochar. Soil available P and K were enhanced two‐ and fivefold, respectively, compared to control and straw‐amended soils. The effects on soil available N were inconsistent, with no significant improvement observed and some treatments indicating possible immobilization. Soil cation exchange capacity (CEC) significantly increased with certain biochar compared to the control but did not differ from straw‐amended soil, with occasional instances where biochar led to lower CEC. Soil available N was higher with biochar application than straw. However, these did not significantly differ from the control, except for biochar produced at 500°C with a 1‐h residence time. Soil available N was notably higher when biochar was surface broadcasted than when thoroughly mixed into the soil. Consequently, this study highlights the influence of biochar pyrolysis conditions on soil nutrient pool, with outcomes also linked to some extent by the application rates and placements, suggesting careful consideration of these management factors for optimal biochar benefit in Luvisols.

## Core Ideas

- The higher temperature biochar products exhibited a notably greater capacity to increase nutrient concentrations.
- Both lower and higher biochar rates augmented soil nutrient pool, each offering distinct advantages.
- Surface broadcasting of biochar showed a trend toward preserving higher soil available N compared to thorough mixing, even though this difference was not statistically significant.
- Biochar notably demonstrated the potential to supplement soil available P and K.

## Plain Language Summary

Biochar, a charcoal‐like material made by heating plant residues, has been widely studied for improving soil health in acidic and degraded soils. However, its effects in fertile soils, such as Luvisols, are underexplored. We conducted two laboratory experiments to explore how biochar production conditions, application rates, and placement affect soil nutrient content. We found that biochar—especially when made at higher temperatures—substantially increased calcium, potassium, magnesium, and sodium in the soil compared to untreated or straw‐amended soils. Biochar also boosted available phosphorus and potassium up to two‐ and fivefold, respectively. In contrast, its effect on nitrogen was less consistent, with some treatments showing no improvement or even signs of nitrogen tie‐up. Interestingly, surface application of biochar tended to maintain more plant‐available nitrogen than thorough mixing. Our findings show that both how biochar is made and how it is applied strongly influence soil nutrient content.

**Abbreviations**
CEC: cation exchange capacity
ICP‐OES: inductively coupled plasma optical emission spectroscopy 
OC: organic carbon
PCA: principal component analysis

## 1 INTRODUCTION

Wheat straw (*Triticum aestivum*), a byproduct of wheat production, is an abundant biomass resource with an annual generation of 529 million tonne worldwide (Govumoni et al., 2013). Among cereal crops in the Canadian prairie, straw production from wheat cultivation is the most abundant, with an average of 25 million tonne generated every year, which the province of Alberta accounted for about 30% of the wheat straw (Sokhansanj et al., 2006). Due to its abundance, renewability, and residual nutrient content, wheat straw is a valuable biomass that can be repurposed to recycle nutrients into arable soils. For instance, the use of straw waste has been effective in elevating soil base cations (K, Ca, Mg, and Na) and available nitrogen (N), as well as reducing reliance on phosphorus (P) mineral fertilization by up to 24% (Cai et al., 2018; Li et al., 2018; Wang et al., 2018; Wei et al., 2021). Thus, returning straw to the soil plays a crucial role in sustainable agriculture and is an effective measure for the resource utilization of crop waste (Sulaiman et al., 2023).

The production of biochar from various biomass materials through pyrolysis has garnered significant interest as an effective soil amendment for its capacity to improve soil fertility and low‐cost feedstock (Gaskin et al., 2008; Gul et al., 2015; Lauricella et al., 2021; Novak et al., 2018; Sulaiman et al., 2024b). Biochar enhances soil nutrient status by increasing the cation exchange capacity (CEC) due to the abundance of negative charge on its surface (Yuan, Xu, Qian et al., 2011), though the extent of this effect depends on feedstock, pyrolysis conditions, soil properties, and the duration of biochar‐soil interaction (Hailegnaw et al., 2019; Manickam et al., 2015). This enhancement of CEC facilitates nutrient retention at soil exchange sites (Novak et al., 2009) and helps maintain the supply of exchangeable calcium (Ca), magnesium (Mg), and K (Yang & Lu, 2022). These nutrients can also become available over time through a biochar‐induced increase in soil pH (Dai et al., 2017; Yang & Lu, 2021). Additionally, biochar can directly release a substantial amount of plant nutrients into the soil, thereby improving nutrient bioavailability (Angst & Sohi, 2013; Hailegnaw et al., 2019; Hong & Lu, 2018; Parvage et al., 2013; Wang et al., 2018; Yan et al., 2022), and in some cases, biochar has been shown to enhance soil P availability up to twofold compared with the direct application of the initial feedstock (Lauricella et al., 2021). The application of biochar not only sustains soil fertility but also significantly improves crop growth and production (Jeffery et al., 2011; Major et al., 2010). In addition, biochar properties vary with pyrolysis temperature and residence time (Ippolito et al., 2020; Wang et al., 2020). For example, the K content of bamboo biochar increased with longer residence time at 400°C and with increasing temperature up to 600°C (Hien et al., 2021), whereas N content in rice straw and canola stalk biochars declined as temperatures rose from 250°C to 650°C (Yang & Lu, 2021). Therefore, identifying optimal production conditions tailored to specific feedstocks and applications is essential for maximizing biochar effectiveness.

Biochar research has predominantly focused on soils with clear constraints, such as acidity, nutrient depletion, or poor structure, where positive effects are most evident (Jeffery et al., 2017). In contrast, outcomes in fertile soils with adequate nutrient status, such as Luvisols, tend to be more nuanced, partly because these soils generally receive greater fertilizer inputs, leaving less room for additional benefits from biochar (Ippolito et al., 2012; Knoblauch et al., 2021; Yao et al., 2025; Ye et al., 2020). For instance, Yao et al. (2025) reported that nearly two‐thirds (66%) of biochar studies in China, based on 104 publications across 22 provinces, were conducted on acidic soils, while Ye et al. (2020) found that 107 studies globally focused on acidic or degraded soils compared with only 41 studies on relatively fertile soils. Similarly, Jeffery et al. (2017) and Lévesque et al. (2020) highlighted that fertile soils such as Luvisols remain underrepresented in biochar research, with only one location worldwide included in each analysis. This underrepresentation likely reflects the perception that management interventions are less urgently required in Luvisols due to their relatively fertile nature. However, given their wide distribution and essential role in sustaining intensive agricultural production across temperate regions (Daly & Hernandez‐Ramirez, 2020; Ejigu et al., 2023; Martyniuk et al., 2019; Obalum et al., 2019), there is a pressing need to investigate biochar–soil interactions in Luvisols.

Biochar application can alter nutrient dynamics in Luvisols by enhancing the availability and retention of key elements such as N and P, thereby increasing the nutrient supply for plant growth (Alburquerque et al., 2014; Kammann et al., 2012). Furthermore, the magnitude of nutrient modification in fertile soils depends on both the application rate and the placement method, with higher rates producing more pronounced effects, and incorporation into the soil promoting stronger responses than surface application (Jílková, 2023; Yan et al., 2022). To address these dynamics in site‐specific Luvisols, the present study evaluates the influence of wheat straw‐derived biochar on soil nutrient modification through controlled laboratory incubations. This experimental design eliminates confounding external factors, such as variable weather conditions, to generate robust insights. We hypothesize that (i) biochar produced at higher pyrolysis temperatures will possess greater inherent nutrient content, leading to stronger enhancement of the soil nutrient pool compared with biochar produced at lower temperatures, and (ii) higher application rates and soil incorporation will promote greater enrichment of the soil nutrient pool than surface application, owing to increased contact between biochar and the soil matrix.

**Core Ideas**

- The higher temperature biochar products exhibited a notably greater capacity to increase nutrient concentrations.
- Both lower and higher biochar rates augmented soil nutrient pool, each offering distinct advantages.
- Surface broadcasting of biochar showed a trend toward preserving higher soil available N compared to thorough mixing, even though this difference was not statistically significant.
- Biochar notably demonstrated the potential to supplement soil available P and K.

## 2 MATERIALS AND METHODS

### 2.1 Soil

The present soil (8‐ to 20‐cm depth increment) was collected from the University of Alberta Breton Research Station (53°05′22″ N, 114°26′27″ W). The soil is classified as Gray Luvisols according to the Canadian System of Soil Classification (AGRASID, 2015), equivalent to Boralf and Albic Luvisols based on the USDA Soil Classification System and FAO World Reference Base for Soil, respectively (Lavkulich & Arocena, 2011). The collected soil was air‐dried, sieved (<2 mm), and composited to a homogenized sample to ensure uniformity between different portions of the sieved soil and stored at room temperature before the experiment. A subsample of the composite soil was analyzed for initial soil properties (Table 1).

**TABLE 1:** Classification and initial properties of the study soil.

Canadian classification	Gray Luvisols
FAO classification	Albic Luvisols^a^
USDA classification	Boralf^a^
pH (1:2 H_2_O)	6.10 ± 0.03
Total organic carbon (%)	1.93 ± 0.08
Total N (%)	0.08 ± 0.01
**Available N (mg kg** ^**−1**^ **soil)**	
NH_4_^+^	8.73 ± 0.09
NO_3_^−^	16.19 ± 0.17
Available P (mg kg^−1^ soil)	9.23 ± 0.12
Available K (mg kg^−1^ soil)	178.30 ± 1.13
**Exchangeable base cation (cmol kg** ^**−1**^ **soil)**	
Exchangeable K	0.64 ± 0.04
Exchangeable Ca	14.64 ± 0.86
Exchangeable Mg	2.80 ± 0.17
Exchangeable Na	0.19 ± 0.01
Cation exchange capacity (cmol kg^−1^ soil)	23.41 ± 0.04
Soil texture	Loam
% Sand	34.32 ± 0.64
% Silt	41.99 ± 0.93
% Clay	23.70 ± 0.39

*Note*: Values represent mean ± standard error of four replicates.

^a^ Lavkulich and Arocena (2011).

### 2.2 Biochar production and analysis

The feedstock used for biochar production was wheat straw (*Triticum aestivum*) collected from the University of Alberta St. Albert Research Station (53°38′0.38″ N, 113°38′7.19″ W). The wheat straw was ground using a laboratory mill model 4 equipped with a 2‐mm sieve (Arthur H. Thomas Company). For the preparation of biochar, batches of approximately 80–90 g of ground wheat straw were placed in ceramic crucibles and pyrolyzed in an enclosed electric furnace (SIB Lindberg). The enclosed furnace chamber creates an oxygen‐limited environment, thereby creating conditions for slow pyrolysis. This setup is similar to those reported in previous studies on crop residue biochar production in a laboratory‐scale furnace without forced airflow (Shi et al., 2019; Wan et al., 2014; Yuan, Xu, Zhang, et al., 2011; Yuan, Xu, Qian, et al., 2011). The furnace was ramped at an average rate of approximately 30°C min^−1^ and held at three different temperatures (350°C, 500°C, and 650°C) for two residence times (1 and 2 h). The resulting biochar was allowed to cool at room temperature and subsequently homogenized. Homogenized subsamples of the biochar were randomly taken for analysis in four replicates. Acronyms for the various biochar included the pyrolysis temperature and residence time; for example, biochar produced at 350°C for 1 h was denoted as BC350‐1. The characteristics of biochar produced under different pyrolysis conditions are listed in Table 2.

**TABLE 2:** Compositions of biochar produced at different pyrolysis conditions.

Parameters	C:N	Yield (%)	pH (1:10)	C (mg g^−1^)	N (mg g^−1^)	P (mg g^−1^)	K (mg g^−1^)	Ca (mg g^−1^)	Mg (mg g^−1^)	Na (mg g^−1^)
Materials										
W	63.94 ± 6.91a	–	6.81 ± 0.05e	433.12 ± 1.72a	7.01 ± 0.74ab	0.59 ± 0.01e	18.24 ± 0.24c	2.53 ± 0.05e	1.17 ± 0.03f	0.11 ± 0.03f
BC350‐1	40.12 ± 1.53bc	12.73 ± 0.47ab	11.28 ± 0.02c	281.62 ± 4.55c	7.05 ± 0.31ab	4.73 ± 0.03c	161.06 ± 5.76ab	20.17 ± 0.24c	9.28 ± 0.09d	0.59 ± 0.01e
BC350‐2	31.84 ± 0.59c	12.16 ± 0.70ab	11.11 ± 0.03d	249.83 ± 4.44d	7.86 ± 0.23a	4.03 ± 0.10d	145.26 ± 7.23b	17.18 ± 0.40d	7.89 ± 0.16e	0.58 ± 0.03e
BC500‐1	49.49 ± 2.93b	13.21 ± 0.50a	11.53 ± 0.02a	297.72 ± 4.82b	6.08 ± 0.37b	5.59 ± 0.10b	174.20 ± 9.31a	22.94 ± 0.55b	10.40 ± 0.21c	0.71 ± 0.01d
BC500‐2	49.05 ± 2.22b	10.46 ± 0.63c	11.35 ± 0.01b	189.93 ± 3.31e	3.89 ± 0.16c	4.91 ± 0.11c	165.26 ± 6.58ab	21.20 ± 0.35c	9.72 ± 0.12d	1.25 ± 0.05c
BC650‐1	66.43 ± 3.61a	11.45 ± 0.41bc	11.40 ± 0.02b	249.48 ± 6.59d	3.78 ± 0.15c	5.98 ± 0.19a	155.28 ± 11.70ab	24.20 ± 0.82b	11.22 ± 0.39b	1.41 ± 0.04b
BC650‐2	46.20 ± 4.24b	9.95 ± 0.40c	11.40 ± 0.02b	161.21 ± 2.75f	3.62 ± 0.47c	6.10 ± 0.15a	153.19 ± 7.90ab	25.89 ± 0.68a	11.95 ± 0.29a	1.60 ± 0.03a
*p* value	<0.001	0.002	<0.001	<0.001	<0.001	<0.001	<0.001	<0.001	<0.001	<0.001
Amount added^a^ with 20 Mg ha^−1^ biochar (kg ha^−1^)	–	–	–	3224.20–5954.38	72.34–157.13	80.60–121.99	2905.26–3484.07	343.64–517.75	157.74–238.90	11.63–32.02

*Note*: Characteristics of wheat straw as initial biochar feedstock were also included for comparison. Different letters in the same column indicate significant difference at α‐critical 0.05. Values reported are mean ± standard error (*n* = 4). The wheat straw (W) biochar (BC) is represented as the pyrolysis temperature and residence time; for example, biochar produced at 350°C with a 1 h residence time is abbreviated as BC350‐1.

^a^ Considering a topsoil layer of 0.20 m is treated and a soil bulk density of 1.42 g cm^−3^.

### 2.3 Experimental procedure

The experimental setup consisted of a laboratory‐scale incubation arranged in a completely randomized design with four replicates at room temperature, where the pots were randomly assigned on the laboratory bench and periodically rotated to equally distribute potential microclimatic variations across treatments. Each incubation unit contained an amount of air‐dry soil equivalent to 60 g oven‐dry soil, which was weighed into a plastic pot (72 mm in height and 46 mm in diameter). Active (non‐sterilized) soils were used to maintain functional microbial communities; however, microbial assays were not performed in this experiment, and their potential role in nutrient dynamics was not directly assessed. Microcosms were closed systems, and no leachate was collected; thus, nutrient losses from leaching were negligible. The equivalent field rate of amendments was calculated considering a soil depth of 0.20 m and a bulk density of 1.42 g cm^−3^ for the Breton plot (Daly et al., 2023). Soil moisture content for all microcosms was maintained at 100% field capacity (35.03 v/v %, Daly et al., 2023) throughout the experiment. This was achieved by adding the equivalent water per pot (∼15 mL) based on the soil bulk density of the plot (1.42 g cm^−3^) and adjusting the total weight of each pot with distilled water every 2 days to compensate for water loss. We chose to conduct the incubation at 100% field capacity to ensure that soil microbial activity and nutrient transformations occurred under non‐limiting moisture conditions, which is a standard approach in controlled laboratory studies aiming to examine the maximum potential effects of soil amendments. All pots were covered with perforated lids to facilitate gaseous exchange while minimizing moisture loss. After concluding the incubation on day 32, all microcosm soil was immediately transferred into individual polyethylene bags, air‐dried at room temperature (∼25°C) without oven‐drying or sieving to preserve their chemical properties as at the end of incubation, homogenized, and stored at ambient temperature before being randomly subsampled for soil analyses.

#### 2.3.1 Experiment 1: Biochar at different pyrolysis conditions

The experiment comprised eight treatments, resulting in a total of 32 soil microcosms. The treatments included an unamended control (CK) soil, soil incorporated with wheat straw (W), and soil amended with six different biochar products: BC350‐1, BC350‐2, BC500‐1, BC500‐2, BC650‐1, and BC650‐2. The soil in each incubation unit was pre‐mixed to simulate soil disturbance during land preparation and was subsequently thoroughly mixed with the biochar and straw treatments at a rate of 400 mg (equivalent field application rate of 20 Mg ha^−1^) using a laboratory spatula. This addition rate might be high but was used to ensure detectable biochar effects and is based on field studies, which indicated its optimality for crop production on loamy soil (Backer et al., 2016; Zhang et al., 2012). The use of this high rate provides mechanistic insights; however, its direct applicability to real‐world practice may be limited, and interpretation should be constrained to the context of laboratory incubations. The control soil was remixed to the same extent as the biochar‐amended soil to ensure consistent soil disturbance across all samples before incubation.

#### 2.3.2 Experiment 2: Biochar placements and application rates

Biochar and straw were applied at the field equivalent rates of 5 and 10 Mg ha^−1^, corresponding to 100 and 200 mg per incubation unit. Each rate was tested under two placement methods—either thoroughly mixed into the soil (M) using a laboratory spatula or placed on the soil surface (L). Due to resource constraints, the biochar products used in this experiment were produced only at 350 and 500°C at a 1 h residence time (BC350 and BC500). The treatments included an unamended CK soil, soil incorporated with wheat straw at two rates and two placement methods (W‐5 M, W‐5L, W‐10 M, and W‐10L), and soil amended with two biochar products at two rates and placement methods (BC350‐5 M, BC350‐5 L, BC350‐10 M, BC350‐10 L, BC500‐5 M, BC500‐5L, BC500‐10 M, and BC500‐10 L), resulting in a total of 13 treatments and 52 soil microcosms. The two application rates were chosen to reflect the feasible amounts that could be applied by farmers, with the two placement methods simulating conventional tillage (M) and no‐till (L) systems.

### 2.4 Laboratory analyses

The pH of biochar and soil was measured with a pH meter (Orion Star A211, Fisher Scientific) by constant agitation in a reciprocal shaker (180 oscillations min^−1^) for 30 min and allowed to equilibrate for 1 h in 1:10 and 1:2 suspension ratio of biochar/soil to milli‐Q water, respectively. Concentrations of total C and N in biochar and soil, as well as soil organic carbon (OC), were determined via dry combustion in an Elemental Analyzer (Thermo FLASH 2000 Organic Elemental Analyzer, Thermo Fisher Scientific Inc.). Total concentrations of biochar P, K, Ca, Mg, and Na were measured with inductively coupled plasma optical emission spectroscopy (ICP‐OES) (Thermo iCAP6300 Duo, Thermo Fisher Corp.) following HNO_3_‐assisted digestion in a closed‐vessel microwave. Soil available N (NH_4_^+^ and NO_3_^−^) was extracted with 50 mL of 2 M KCl, while soil available P and K were extracted using the modified Kelowna method in a reciprocal shaker (180 oscillations min^−1^) for 30 min (Ashworth & Mrazek, 1995). Available N and P in the extract were quantified by colorimetry (Thermo Gallery Plus Beermaster Autoanalyzer, Thermo Fisher Scientific), while available K was determined by ICP‐OES (Thermo iCAP6300 Duo, Thermo Fisher Corp.). For the analysis of soil CEC, the soil sample was saturated with a buffered 1 M NH_4_OAc solution and filtered through Whatman 42 paper (2.5 µm fine porosity, Whatman International, Maidstone, England). The soil samples were then leached with an unbuffered 1 M KCl solution. Soil exchangeable base cations (K, Ca, Mg, and Na) were analyzed from the NH_4_OAc leachate by ICP‐OES. The concentration of NH_4_^+^ in the final KCl leachate was analyzed by colorimetry and reported as the soil CEC (Hendershot et al., 1993).

### 2.5 Statistical analyses

Statistical analyses were conducted using RStudio software version 4.2.764 (Posit Team, 2024) with a 5% significance level (*p* ≤ 0.05). The data were assessed for normality using the Shapiro–Wilk test and for homoscedasticity using the Levene test. Data transformations were applied as necessary. A one‐way analysis of variance (ANOVA) was employed on linear models developed for all measured variables, with biochar products and soil treatments in Experiment 1 as the fixed effects. A two‐way ANOVA was conducted for all measured variables in Experiment 2, with application rates and placement methods as the fixed effects. Post hoc analysis was performed via Fisher's least significant difference test in the *agricolae* package (de Mendiburu, 2021). Principal component analysis (PCA) was performed to explore the relationships between biochar traits and soil nutrient responses and to assess the influences of application rate and placement on these responses. To allow comparability among variables measured in different units, all numeric data were centered and scaled prior to ordination. PCA was conducted using the prcomp function in the *FactoMineR* package (Husson et al., 2020), while visualization and interpretation of the ordinations were carried out using the *factoextra* package (Kassambara & Mundt, 2020). Interpretation focused on the first two principal components (PC), which explained the greatest proportion of variance in the dataset. In the resulting biplots, sample points represented the treatment factors (pyrolysis temperature, application rate, and placement), while soil and biochar variables were represented as loading vectors.

## 3 RESULTS

### 3.1 Changes in properties of biochar‐added soil as affected by the different pyrolysis conditions of biochar

**FIGURE 1:**
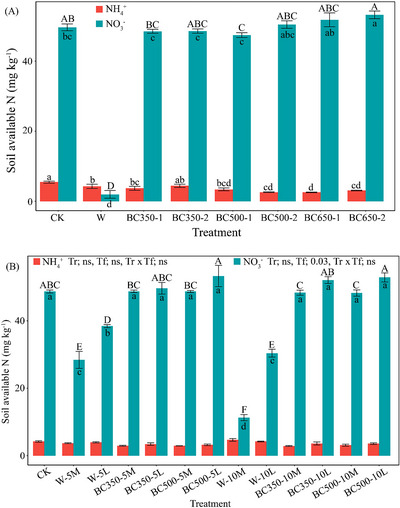
The concentration of soil available N (NH_4_^+^ and NO_3_^−^) at the end of incubation as affected by biochar application across various pyrolysis conditions in Experiment 1 as well as rates and placements in Experiment 2. Different lowercase letters indicate significant differences between treatments for NH_4_^+^ and NO_3_^−^, while uppercase letters denote significant differences in total available N. Statistical significance was assessed at *p* ≤ 0.05, and values are presented as mean ± standard error (*n* = 4). (A) In Experiment 1, treatments are abbreviated by wheat straw (W), biochar type (BC), and pyrolysis conditions (e.g., BC350‐1 for biochar produced at 350°C with a 1 h residence time). (B) In Experiment 2, treatments were assessed by application rates (5 or 10 Mg ha^−1^) and placements (mixed, M; surface, L), and abbreviated by wheat straw (W) biochar type (BC), rate, and placement (e.g., BC350‐5L represents biochar produced at 350°C with a 1 h residence time, applied at 5 Mg ha^−1^ on the soil surface). Statistical outcomes for rate (Tr), placement (Tf), and their interaction (Tr × Tf) were determined by two‐way analysis of variance (ANOVA), with non‐significant differences denoted as ns.

**FIGURE 2:**
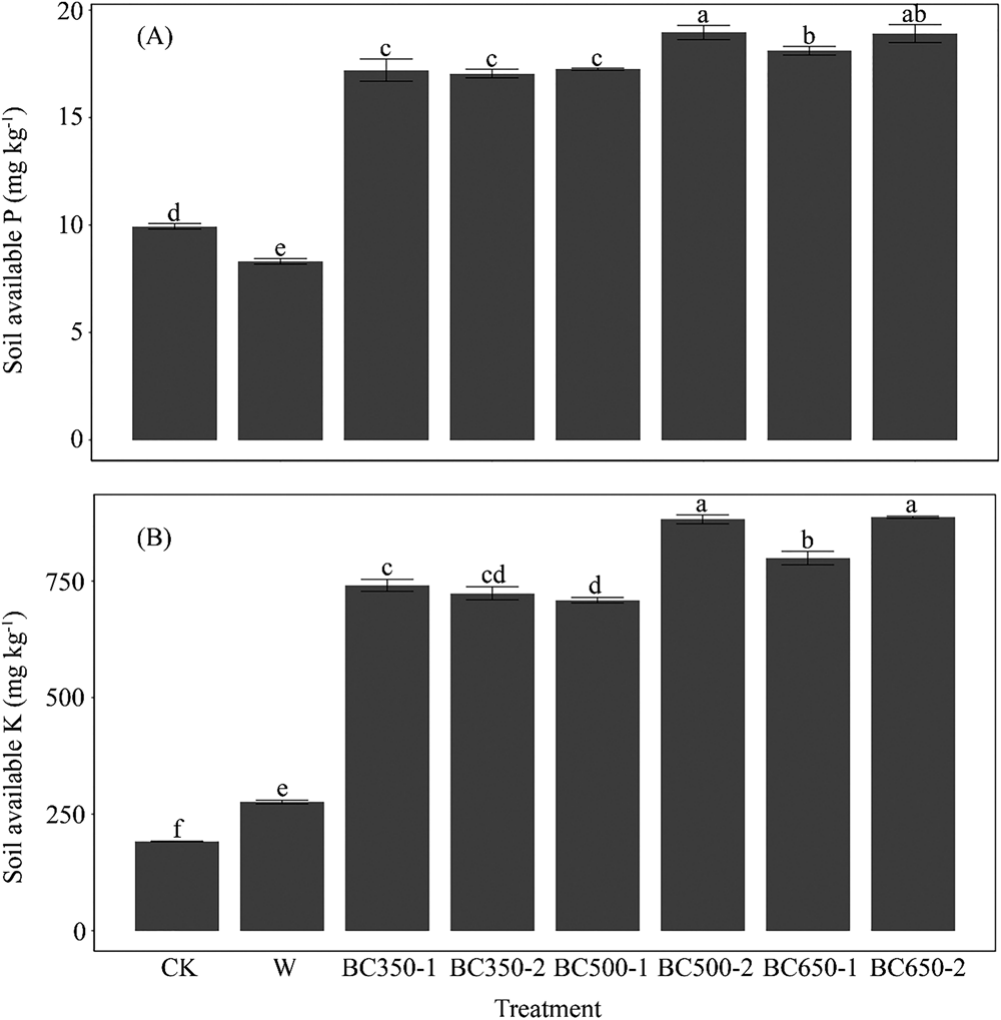
The concentrations of soil (A) available P and (B) available K at the end of incubation as affected by biochar application across various pyrolysis conditions in Experiment 1. Different letters indicate significant differences between treatments at *p* ≤ 0.05. Values reported are mean ± standard error (*n* = 4). The treatments are abbreviated by the wheat straw (W) biochar type (BC) and the pyrolysis conditions. For instance, the application of biochar produced at 350°C with a 1‐h residence time is denoted as BC350‐1. CK, unamended control.

Biochar‐amended soils have significantly higher soil pH by 0.31–0.44 units compared to the control at the end of the incubation (F_7, 24_ = 18.58, *p* < 0.001, *η* ^2^ = 0.84; Table 3). However, these pH increments were not significantly different from the effect of wheat straw addition. The concentration of soil available N was significantly higher (F_7, 24_ = 192.06, *p*  < 0.001, *η* ^2^ = 0.98) in biochar‐amended soils than in the control (Figure 1A). However, biochar‐added soils generally resulted in lower available N compared to the control, albeit this difference was not statistically significant. When examining the individual components of available N, NH_4_^+^ content was significantly lower in biochar treatments relative to the control (F_7, 24_ = 6.82, *p* < 0.001, *η* ^2^ = 0.67), except for soil treated with BC350‐2. Relative to straw‐amended soil, NH_4_^+^ content was significantly lower in soils treated with BC350‐1, BC350‐2, and BC500‐1. Conversely, NO_3_^−^ content was significantly (F_7, 24_ = 244.33, *p *< 0.001, *η* ^2^ = 0.99) higher in soil incorporated with BC650‐2 compared to the control. The NO_3_^−^ content was also significantly greater in biochar‐amended soils than in straw‐amended soil. Biochar application significantly increased soil available P (Figure 2A) by up to twofold compared to the control and straw‐amended soils (F_7, 24_ = 206.98, *p *< 0.001, *η* ^2^ = 0.98). The highest soil available P concentration was observed in soils treated with BC500‐2 and BC650‐2. Although wheat straw addition significantly increased soil available K relative to the unamended control, the effect was markedly enhanced by the addition of all biochar products, with increases of up to fivefold (F_7, 24_ = 802.61, *p *< 0.001, *η* ^2^ = 1.00; Figure 2B). The highest soil available K concentration was observed in soils treated with BC500‐2 and BC650‐2. The OC concentrations were elevated in all treatments compared to the unamended control; however, significant (F_7, 24_ = 4.60, *p* ≤ 0.05, *η* ^2^ = 0.57) increases were only apparent in soils amended with straw, BC350‐1, BC350‐2, and BC500‐1. The OC content did not significantly differ between biochar‐amended and straw‐amended soils. Among soil exchangeable cations, Ca was dominant, with significantly (F_7, 24_ = 7.98, *p* < 0.001, *η* ^2^ = 0.70) higher values at the end of incubation in soils treated with BC350‐1, BC350‐2, BC500‐1, and BC500‐2 compared to the unamended control. Biochar addition resulted in significantly higher exchangeable Ca content than straw‐added soil, apart from soils treated with BC350‐1, BC650‐1, and BC650‐2. The concentrations of soil exchangeable K, Mg, and Na were significantly higher with biochar addition relative to both the unamended control and straw‐amended soils. Notably, soil exchangeable K was significantly higher (F_7, 24_ = 899.65, *p* < 0.001, *η* ^2^ = 1.00) with the addition of biochar produced at a prolonged residence time (2 h) and higher temperature than at a shorter residence time (1 h) and lower temperature, with the highest soil exchangeable K observed in soil treated with BC650‐2. Similarly, the highest soil exchangeable Na was observed in soil treated with BC650‐2 (F_7, 24_ = 23.35, *p* < 0.001, *η* ^2^ = 0.87). The addition of biochar produced with a 2 h residence time had significantly higher (F_7, 24_ = 60.72, *p *< 0.001, *η* ^2^ = 0.95) soil exchangeable Mg compared to a 1 h residence time at 350 and 500°C (Table 3). Soil CEC was significantly (F_7, 24_ = 4.67, *p* ≤ 0.05, *η* ^2^ = 0.58) increased to 25.00, 25.14, and 24.96 cmol kg**^−^** ^1^ in soils treated with BC350‐2, BC500‐1, and BC500‐2, respectively, relative to the unamended control (23.84 cmol kg**^−^** ^1^). However, these values did not significantly differ from straw‐added soil (25.16 cmol kg**^−^** ^1^). In certain cases, biochar application significantly lowered soil CEC, such as in soils treated with BC350‐1, BC650‐1, and BC650‐2, compared to straw‐added soil (Table 3).

**TABLE 3:** Effect of biochar produced at various pyrolysis conditions on pH, organic carbon (OC), and cation exchange performance of soil.

Parameters	pH	OC	CEC	Exch. K	Exch. Ca	Exch. Mg	Exch. Na
Treatments	1:2	%	cmol kg^−1^				
CK	5.67 ± 0.02c	1.87 ± 0.03a	23.84 ± 0.05c	0.63 ± 0.02 g	14.05 ± 0.07d	2.66 ± 0.02e	0.19 ± 0.001e
W	6.03 ± 0.03ab	2.07 ± 0.02bc	25.16 ± 0.30a	0.79 ± 0.004f	14.31 ± 0.13 cd	2.74 ± 0.01d	0.19 ± 0.003e
BC350‐1	5.98 ± 0.03b	2.47 ± 0.30c	23.98 ± 0.45c	1.83 ± 0.02e	14.52 ± 0.13bc	3.03 ± 0.03c	0.20 ± 0.001d
BC350‐2	5.98 ± 0.02b	2.25 ± 0.10c	25.00 ± 0.34ab	1.93 ± 0.04d	14.87 ± 0.07a	3.11 ± 0.03ab	0.21 ± 0.002bc
BC500‐1	6.02 ± 0.04b	2.27 ± 0.15c	25.14 ± 0.13a	1.84 ± 0.02e	14.78 ± 0.08ab	3.07 ± 0.02bc	0.20 ± 0.001d
BC500‐2	6.11 ± 0.04a	1.98 ± 0.03ab	24.96 ± 0.25ab	2.24 ± 0.03b	14.76 ± 0.12ab	3.15 ± 0.03a	0.20 ± 0.002 cd
BC650‐1	5.99 ± 0.04b	1.96 ± 0.07ab	24.18 ± 0.33bc	2.06 ± 0.02c	14.23 ± 0.17 cd	3.05 ± 0.03bc	0.21 ± 0.002b
BC650‐2	6.04 ± 0.01ab	1.92 ± 0.02ab	23.44 ± 0.44c	2.33 ± 0.01a	14.22 ± 0.06 cd	3.11 ± 0.01ab	0.22 ± 0.001a
*p* value	<0.001	0.002	0.002	<0.001	<0.001	<0.001	<0.001

*Note*: Different letters in the same column indicate significant differences at α‐critical 0.05. Values reported are mean ± standard error (*n* = 4). The wheat straw (W) biochar (BC) is represented as the pyrolysis temperature and residence time; for example, biochar produced at 350°C with a 1 h residence time is abbreviated as BC350‐1.

Abbreviations: CEC, cation exchange capacity; CK, unamended control; Exch., exchangeable.

**FIGURE 3:**
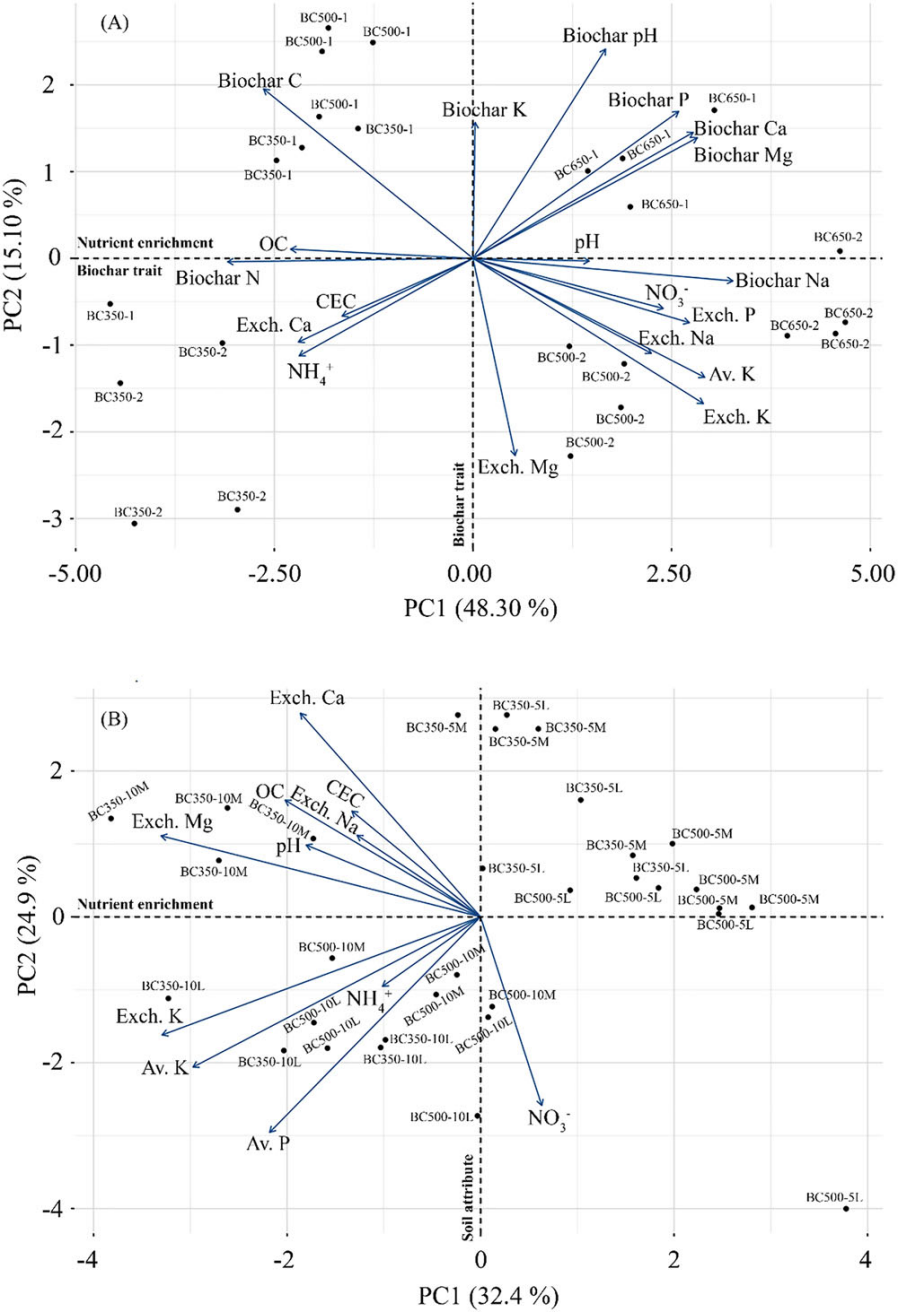
Principal component analysis (PCA) ordination showing the relationships between soil responses and biochar factors in two independent experiments. (A) Experiment 1: soil responses in relation to biochar characteristics across pyrolysis conditions; each point represents a distinct biochar product (e.g., BC350‐1 = biochar produced at 350 °C for 1 h). (B) Experiment 2: soil responses in relation to biochar application strategies, including placement (surface, L; mixed, M) and rate (5 vs. 10 Mg ha^−1^); each point represents a treatment combination (e.g., BC350‐5L = biochar produced at 350°C for 1 h, applied at 5 Mg ha^−1^ to the soil surface). Vectors indicate soil properties contributing most strongly to the principal components. CEC, cation exchange capacity; OC, organic carbon.

Further exploration using PCA revealed that the first two principal components accounted for 63.40% of the total variance (Figure 3A). Soil nutrient parameters loaded strongly on PC1, representing a gradient of overall available nutrients and chemical fertility. Biochar traits such as P, Mg, and Na also loaded heavily on PC1, indicating that biochar products enriched in these elements directly contribute to soil nutrient enhancement. Biochar traits were also distributed along PC2, highlighting that biochar influences soil properties not only via direct nutrient supply but also through indirect mechanisms, such as via modulation of soil pH. Notably, biochar Ca and exchangeable Ca were positioned in opposite quadrants of the biplot, suggesting a negative correlation. Likewise, the separation of biochar N and NH_4_^+^ with NO_3_^−^ loadings underscores contrasting associations between biochar N with NH_4_^+^ and NO_3_^−^ in the soil system. Moreover, the distribution of biochar P and K far from soil available P, exchangeable, and available K in the biplot suggests that the P and K contents of biochar alone are not the primary driver of soil P and K dynamics. Additionally, biochars produced at higher temperatures clustered along the nutrient‐enrichment gradient of PC1, highlighting their significant influence on soil nutrient dynamics.

### 3.2 Changes in properties of biochar‐added soil as affected by application rates and placement method of biochar

**FIGURE 4:**
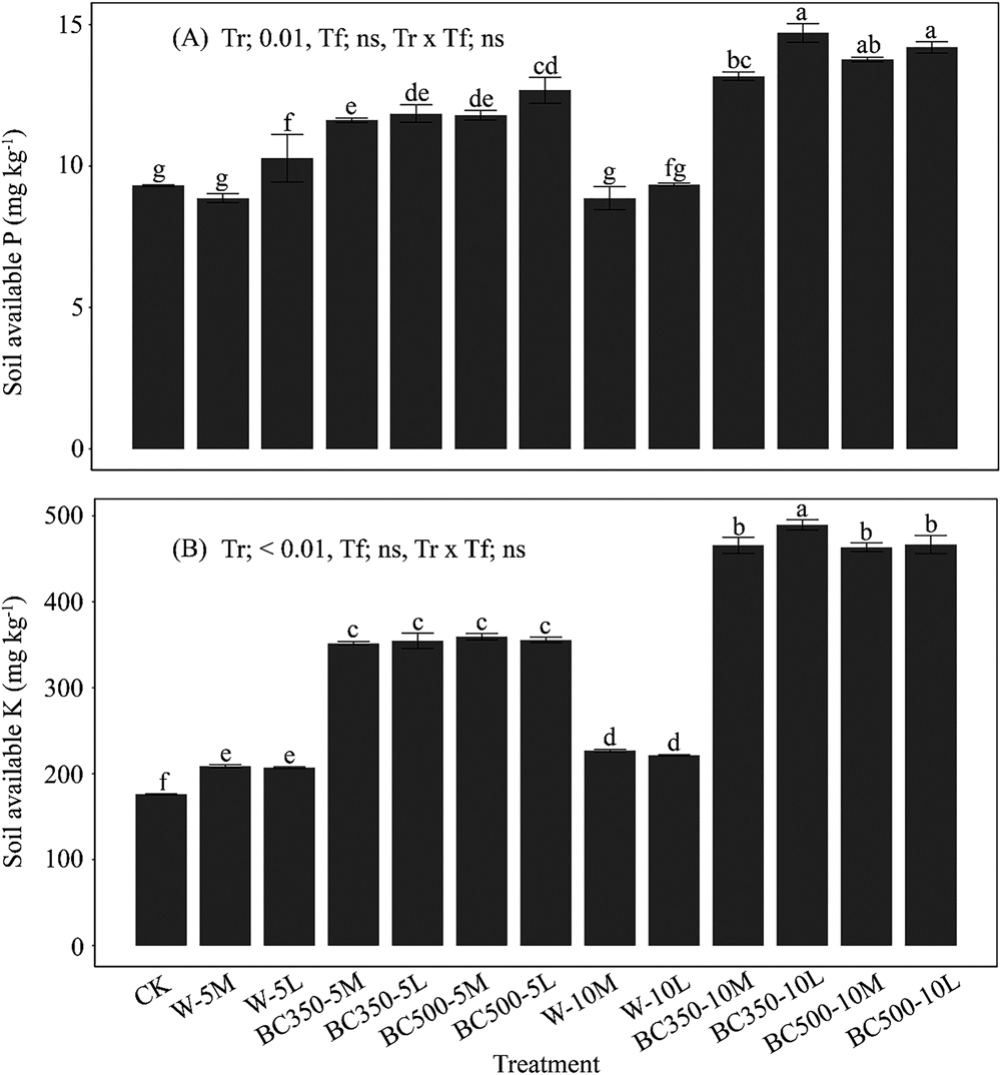
The concentrations of soil (A) available P and (B) available K at the end of incubation as affected by biochar application rates (5 or 10 Mg ha^−1^) and placements (mixed, M or surface, L) in Experiment 2. Values presented are mean ± standard error (*n* = 4). Different letters indicate significant differences between treatments. Statistical significance outcomes are indicated as Tr for application rate, Tf for application placement, and Tr × Tf for the interaction between application rate and placement, based on *p* ≤ 0.05 using a two‐way analysis of variance (ANOVA). Non‐significant differences are denoted with ns. Treatments are abbreviated using a notation system specifying wheat straw (W), biochar type (BC), application rate, and form. For example, biochar produced at 350°C with a 1 h retention time, applied at a rate of 5 Mg ha^−1^ broadcast on the soil surface, is denoted as BC350‐5L. CK, unamended control.

The effect of biochar application rate was significant for most soil properties. The contents of available P, available K, OC, exchangeable K, exchangeable Mg, and soil pH were, on average, 25%–31% (F_2, 47_ = 5.08, *p* < 0.01, *η* ^2^ = 0.17), 100%–178 % (F_2, 47_ = 10.47, *p* < 0.001, *η* ^2^ = 0.31), 8%–22 % (F_2, 47_ = 10.77, *p* < 0.001, *η* ^2^ = 0.31), 58%–103% (F_2, 47_ = 9.45, *p* < 0.001, *η* ^2^ = 0.29), 5%–3 7% (F_2, 47_ = 5.83, *p* < 0.01, *η* ^2^ = 0.20), and 2%–5 % (F_2, 47_ = 17.20, *p* < 0.001, *η* ^2^ = 0.40) higher, respectively, than in the control at the end of incubation (Table 4; Figure 4). The high application rate had stronger effects than the low application rate. Notably, the contents of available P, available K, exchangeable K, and exchangeable Mg were, on average, 12%–24%, 29%–38%, 24%–28%, and 3%–4% higher with the high rate compared to the low rate at the end of incubation. These values in biochar‐added soil for available P, available K, exchangeable K, and exchangeable Mg were also significantly higher than the corresponding values in wheat‐added soil. There was no significant rate effect (*p* > 0.05) for soil available N, CEC, exchangeable Ca, and Na in all treatments.

**TABLE 4:** Effect of biochar on pH, organic carbon (OC), and cation exchange attributes of soil at the end of incubation as affected by application placements (broadcast on the soil surface as L, or thoroughly mixed into the soil as M) and rates (5 or 10 Mg ha^−1^).

Parameters	pH (1:2)	OC (%)	CEC (cmol kg^−1^)	Exch. Na (cmol kg^−1^)	Exch. K (cmol kg^−1^)	Exch. Ca (cmol kg^−1^)	Exch. Mg (cmol kg^−1^)
Treatments							
CK	5.61 ± 0.02g	1.72 ± 0.04e	22.85 ± 0.38f	0.19 ± 0.0001a	0.62 ± 0.002f	14.39 ± 0.07ab	2.68 ± 0.01d
W‐5 M	5.81 ± 0.01bcde	1.82 ± 0.03de	24.04 ± 0.25abcd	0.20 ± 0.005abc	0.70 ± 0.02de	14.48 ± 0.08ab	2.70 ± 0.03cd
W‐5L	5.73 ± 0.03ef	1.82 ± 0.03de	24.77 ± 0.25ab	0.19 ± 0.001ab	0.67 ± 0.01e	14.43 ± 0.04ab	2.68 ± 0.01d
W‐10 M	5.95 ± 0.01a	2.03 ± 0.14abc	24.66 ± 0.26abc	0.19 ± 0.001abc	0.69 ± 0.01de	14.19 ± 0.12bc	2.68 ± 0.01d
W‐10L	5.88 ± 0.02abc	2.00 ± 0.02abc	24.97 ± 0.24a	0.19 ± 0.002abc	0.71 ± 0.01d	14.35 ± 0.04ab	2.73 ± 0.01cd
BC350‐5 M	5.82 ± 0.02bcd	2.07 ± 0.03ab	23.91 ± 0.41bcde	0.20 ± 0.01c	0.97 ± 0.01c	14.50 ± 0.18ab	2.81 ± 0.02b
BC350‐5L	5.80 ± 0.02cde	1.98 ± 0.06abcd	23.56 ± 0.05def	0.20 ± 0.001bc	0.99 ± 0.01c	14.46 ± 0.10ab	2.81 ± 0.02b
BC350‐10 M	5.89 ± 0.04ab	2.09 ± 0.03ab	23.80 ± 0.83cde	0.23 ± 0.02d	1.24 ± 0.04a	14.53 ± 0.12a	2.91 ± 0.02a
BC350‐10L	5.79 ± 0.05def	2.12 ± 0.02a	23.66 ± 0.11def	0.20 ± 0.01abc	1.25 ± 0.03a	13.95 ± 0.10cd	2.81 ± 0.02b
BC500‐5 M	5.77 ± 0.02def	1.94 ± 0.07bcd	23.18 ± 0.21def	0.20 ± 0.003abc	0.95 ± 0.01c	13.81 ± 0.04d	2.71 ± 0.01cd
BC500‐5L	5.71 ± 0.02f	1.87 ± 0.10cde	23.08 ± 0.19ef	0.20 ± 0.01c	0.98 ± 0.01c	13.77 ± 0.17d	2.74 ± 0.03c
BC500‐10 M	5.74 ± 0.03def	2.00 ± 0.06abc	23.27 ± 0.15def	0.19 ± 0.001abc	1.18 ± 0.01b	13.92 ± 0.10cd	2.82 ± 0.02b
BC500‐10L	5.80 ± 0.04cde	1.93 ± 0.04bcd	23.31 ± 0.10def	0.19 ± 0.004abc	1.21 ± 0.01ab	13.96 ± 0.17cd	2.82 ± 0.04b
**Significance level**							
Rate	<0.001	<0.001	0.07	0.20	<0.001	0.37	<0.001
Form	0.05	0.35	0.75	0.36	0.88	0.40	0.75
Rate × form	0.80	0.76	0.96	0.68	0.89	0.65	0.72

*Note*: Different letters on the same column indicate significant differences at α‐critical 0.05. Values reported are mean ± standard error (*n* = 4). The treatments are abbreviated by the wheat straw (W) biochar type (BC), rate, and application form. For example, the application of biochar produced at 350 with a 1 h residence time at a rate of 5 Mg ha^−1^ broadcast on the soil surface is abbreviated as BC350‐5L.

Abbreviations: CEC, cation exchange capacity; CK, unamended control; Exch., exchangeable.

Although the effect of biochar placement methods was not significant (*p* > 0.05) for soil available P, available K, OC, exchangeable K, Ca, Mg, and Na, and CEC at the end of incubation, there was, on average, a 5% numerically higher value for soil pH (F_1, 47_ = 4.04, *p* ≤ 0.05, *η* ^2^ = 0.05) when BC350‐10 was mixed into the soil (to simulate no till) rather than placed on the soil surface (to simulate conventional tillage) compared to the unamended control at the end of incubation (Table 4). Conversely, soil available N was, on average, 5%–7% higher (F_1, 47_ = 4.93, *p* ≤ 0.05, *η* ^2^ = 0.09) when biochar was placed on the soil surface rather than mixed into the soil (Figure 1B). This was observed in the BC350‐10, BC500‐5, and BC500‐10 treatments, although these values did not statistically differ from the unamended control. Nevertheless, all biochar‐amended soils had significantly higher available N (51.23–56.52 mg kg**^−^** ^1^) than wheat‐amended soils (15.96–42.38 mg kg**^−^** ^1^).

The PCA revealed a distinct separation of treatments largely governed by application rate (Figure 3B). The first two components explained 57.30% of the total variance, with PC1 (32.40%) representing a nutrient enrichment axis strongly associated with available P, available K, exchangeable K, and Mg, while PC2 (24.90%) reflected broader soil chemical attributes linked to CEC, OC, and pH. Treatments receiving 10 Mg ha^−1^ biochar clustered closely with nutrient vectors, signifying marked enrichment and fertility enhancement, whereas 5 Mg ha^−1^ treatments were positioned more distantly, consistent with weaker nutrient responses. Biochar placement (mixed vs. surface‐applied) produced no clear separation along either axis, although slight dispersion among the 10 Mg ha^−1^ treatments hinted at minor placement effects on soil–nutrient interactions.

## 4 DISCUSSION

### 4.1 Effect of biochar produced at various pyrolysis conditions

Due to the significantly elevated concentrations of Na, K, Ca, and Mg in biochar compared to the wheat straw (Table 2), biochar generally enhanced soil exchangeable cations to varying degrees (Table 3). The cations contained in biochar are weakly held via electrostatic forces, allowing their dissolution into the soil solution and elevating their concentrations (Chintala et al., 2014). It was interesting that, while biochar addition elevated soil exchangeable Ca relative to the unamended control, the increase was only significant in soils added with low‐ and medium‐temperature biochar products, despite the higher Ca content in the high‐temperature biochar. In the case of low‐ and medium‐temperature biochar, the release of Ca from biochar likely contributed to the observed increase in soil exchangeable Ca concentration. Conversely, in soils added with high‐temperature biochar, the observed decrease in exchangeable Ca and the negative alignment of biochar Ca with soil exchangeable Ca in the biplot suggest that elevated Ca in biochar does not necessarily result in soil Ca enhancement (Figure 3A). This pattern may indicate a Ca‐K exchange reaction, in which Ca in the soil is displaced by K released from biochar (Limwikran et al., 2018). Confirming this hypothesis would require targeted ion exchange or solution‐phase studies, which are recommended for future research.

Biochar addition to soil greatly enhanced soil available K by a considerable margin (Figure 2B). This biochar‐mediated increase in soil‐available K was five times higher than the unamended control and three times more prominent than the direct application of straw. Due to the high solubility of K‐containing salts formed during pyrolysis (Angst & Sohi, 2013), the significant increase in soil‐available K could be derived from the direct release of added biochar K, which had a substantially higher K concentration than straw (Table 2). The released K could also occupy soil exchange sites, enhancing soil exchangeable K (Table 3), which can be released into the soil solution and become available. Previous studies have also shown that biochar addition increased the K concentration in soil (Novak et al., 2018; Rasuli et al., 2022). Furthermore, increasing the temperature of biochar products further substantiates the increase in soil K concentration (Rasuli et al., 2022), with high‐ and medium‐temperature biochar products (BC500‐2 and BC650‐2) resulting in higher soil available and exchangeable K compared to their lower temperature counterpart (BC350‐2). This apparent incongruency is attributed to the decomposition of organically bound K (e.g., R‐COOK) at higher temperatures, making the formed K easier to release into the soil solution than the original organically bound K (Chen et al., 2023). However, the spatial separation of soil available K and exchangeable K from biochar K in the biplot (Figure 3A) suggests that direct release from biochar is not the only mechanism underlying the observed enrichment. Additionally, biochar addition may stimulate the activity of K‐dissolving bacteria that can solubilize K‐bearing minerals in the soil and convert the insoluble K to available forms (Wang et al., 2018). Interestingly, the K content of the present biochar products (145.26–174.20 mg g^−1^) was higher than biochar derived from common agricultural residues reported in the literature, such as cow manure biochar (2.60–3.00 mg g^−1^, 300–500°C; Beheshti et al., 2017), wheat chaff biochar (18.40 mg g^−1^, 550°C; Lauricella et al., 2021), rice straw biochar (36.10–54.20 mg g^−1^, 300–650°C; Yang & Lu, 2021), and poultry litter biochar (74.00 mg g^−1^, 700°C; Cantrell et al., 2012).

Our data indicated that biochar application increased soil available P by up to twofold compared with both the unamended control and straw addition (Figure 2A). This stronger effect is consistent with the substantially higher P content in biochar, seven to ten‐fold greater than straw across pyrolysis conditions (Table 2)—reflecting the enrichment of biochar P and its transformation into more soluble inorganic forms during pyrolysis (Lauricella et al., 2021; Sulaiman et al., 2024a; Xu et al., 2016; Yang & Lu, 2021). The temperature effect was particularly evident: biochar produced at 650°C significantly increased soil available P compared to lower temperature products (350°C and 500°C at a 1 h residence time), while at 500°C, longer residence time (2 h) also enhanced available P by approximately 2 mg kg**^−^** ^1^ (equivalent to 5.68 kg P ha**^−^** ^1^) despite a slight decline in biochar total P content. This increase coincided with a small but significant rise in soil pH (Table 3), suggesting that both direct P dissolution from biochar and pH‐driven solubilization of soil P contributed to the observed effect (Gustafsson et al., 2012; Hong & Lu, 2018; Yang & Lu, 2021). In addition to higher intrinsic P content, biochar produced at elevated temperatures typically displays higher surface area and porous structure that can promote microbial P solubilization (Li et al., 2020; Zhou et al., 2020). The PCA biplot supports these multifaceted mechanisms: Biochar P is relatively distant from soil‐available P along both PC1 and PC2, indicating that direct P release alone does not explain the observed changes (Figure 3A). The segregation suggests that other processes—such as pH‐driven solubilization and microbial mineralization—also contribute to P mobilization, especially at higher pyrolysis temperatures.

Although biochar contained similar or even lower N content relative to straw (Table 2), our results did not indicate a significant overall enhancement of soil available N compared to the unamended control. The absence of significant differences between most biochar treatments and the unamended control indicates the likelihood of N immobilization, contributing to the lack of enhancement in available N. For example, BC500‐1 immobilized up to 5 mg N kg**^−^** ^1^ (equivalent to 14.2 kg N ha**^−^** ^1^). The low N content and high C:N ratio (>20) of our biochar products likely stimulated microbial assimilation of inorganic N, rendering the N unavailable (Chan & Xu, 2009). However, our results also showed that the concentration of available N was significantly higher in biochar‐added soil than in straw‐added soil (Figure 1A). This was mainly reflected in lower NH_4_^+^ and correspondingly higher NO_3_^−^ concentrations in biochar‐added soil, indicating biochar‐induced nitrification (Clough et al., 2013). This pattern is also reflected in the biplot (Figure 3A), where the positioning of NO_3_^−^ in the opposite quadrant to biochar N and soil NH_4_^+^ suggests a distinct pathway of N transformation through nitrification. Therefore, the intermediate N levels in biochar treatments compared to straw treatment likely reflect a balance between rapid biochar‐induced nitrification and microbial N immobilization, rather than a net increase in available N. It should also be noted that potential NH_3_ volatilization, particularly from high‐pH biochar treatments, was not measured in this study, which could equally affect the measured NH_4_^+^ concentration. In addition, incubation at 100% field capacity may have promoted the formation of anaerobic microsites, creating conditions that could enhance denitrification and result in N_2_O emissions. Thus, pristine biochar, even when applied at relatively high N input (equivalent to 72.34–157.13 kg N ha**^−^** ^1^), does not provide a reliable strategy for enhancing soil‐available N. Nevertheless, we still consider this lack of difference important because the N immobilization capacity of biochar could help minimize the loss of N from the soil system to the environment when applied alongside chemical N input, given that N fertilizer sole application can result in losses as high as 50%–70 % of the applied N (Ladha et al., 2005).

The observed increases in CEC of some biochar‐amended soils (BC350‐2, BC500‐1, and BC500‐2) compared to the unamended control (Table 3) are consistent with the widely recognized role of negatively charged biochar surfaces in enhancing soil CEC (Gul et al., 2015; Liang et al., 2006; Yuan, Xu, Qian, & Wang, 2011). However, these increases were not consistent across treatments. In certain cases (BC350‐1, BC650‐1, and BC650‐2 soils), biochar application did not significantly alter CEC relative to the control and even resulted in lower values than the straw‐added soil. Limited studies compared soil CEC between straw application and its resulting biochar under similar experimental conditions, but the available literature suggests crop residue biochar produced at various pyrolysis conditions generally increases CEC compared to their initial feedstocks (Yang & Lu, 2021; Yuan, Xu, Qian, & Wang, 2011). Nonetheless, our observation of a decrease in soil CEC with biochar addition—in contrast to the increase with straw—contributes to the literature by illustrating a divergent outcome, which emphasizes that the effect of biochar on CEC is treatment‐ and context‐dependent, rather than universally positive. These contrasting outcomes suggest that factors such as the interaction of humic and fulvic acids of SOM with biochar pores may limit CEC increase under certain conditions (Pignatello et al., 2006), emphasizing the need for caution when generalizing the benefits of biochar for soil CEC.

### 4.2 Effect of application rates and placement method of biochar

The adoption of different cultivation systems, such as conventional tillage and no‐till, leads to spatially distinct amendment additions with varying effects on the nutrient pool. Conventional tillage, involving thorough soil incorporation of amendments, is expected to have a stronger enhancement of the nutrient pool compared to no‐till, where amendments remain on the surface, as previously observed with dissolved soil N (Jílková, 2023). Over time, surface recurrent broadcasting could even lead to the stratification of soil properties or the exposure of added biochar to erosive losses by wind or water (Dai et al., 2017; Sarfaraz et al., 2024; Silva et al., 2015). Nevertheless, the present study found that the effect on soil available N was more pronounced in the simulated no‐till system, particularly in the BC350‐10, BC500‐5, and BC500‐10 soils (Figure 1B). Although these values tended to be higher compared to the control, the differences were not statistically significant. The PCA biplot demonstrated that soil NO_3_^−^ loaded independently along PC1 and displayed little correspondence with biochar rate (Figure 3B), implying that NO_3_^−^ dynamics is less responsive to direct biochar additions and are instead shaped by indirect processes such as microbial‐mediated effect. The proximity of biochar to the soil environment and soil microbes in the simulated conventional tillage system could have induced microbial N immobilization (Chan & Xu, 2009) and re‐adsorption of released N onto the biochar surface (Gao et al., 2019), restricting available N. Instead, biochar placed on the surface could limit these processes altogether, resulting in the greater available N. Nonetheless, the application of biochar in both simulated systems significantly enhanced the soil available N compared to the application of straw by a substantial margin of 10.74–35.41 mg kg**^−^** ^1^ (equivalent to 30.50–100.56 kg N ha**^−^** ^1^), even though enhancements relative to the control were not observed.

Biochar application rate is another crucial factor in altering the nutrient pool, with a high rate (40 Mg ha^−1^) exhibiting a much stronger effect than a lower rate (20 Mg ha^−1^) under controlled experimental conditions in a fertile soil (Jílková, 2023). Our study, which employs lower and more feasible application rates, found a similar rate‐dependent effect, particularly prominent in elevating soil available P and K (Figure 4). Similarly, our PCA biplot demonstrates that biochar application rate exerted a stronger influence than placement on soil nutrient dynamics (Figure 3B), suggesting that nutrient enrichment was primarily achieved through greater biochar inputs. The higher application rate (10 Mg ha^−1^) had a stronger effect than the low application rate (5 Mg ha^−1^) with both biochar types (BC350 and BC500), irrespective of the different simulated cultivation systems. Although the impact of the higher rate (10 Mg ha^−1^) was stronger, the soil benefited from both evaluated rates, which emphasizes the flexibility and adaptability of biochar as a soil amendment. This highlights the detectable benefit of even the low biochar rate on the fertile Luvisols, challenging the assumption that a high biochar rate (>9 Mg ha^−1^) is needed for the beneficial effect on the soil nutrient pool to become apparent in a relatively fertile soil (Lévesque et al., 2020).

### 4.3 Limitations and future work

This study was conducted under controlled laboratory conditions using a single fertile Luvisols sample, with only two biochar application rates (5 and 10 Mg ha^−1^) and two placement methods (mixing and broadcasting), and focused exclusively on wheat straw with a limited range of pyrolysis temperatures and residence times. As biochar effects are influenced by soil properties, management practices, and environmental conditions, the observed results may not directly translate to other soil types, climates, feedstocks, or real‐world farming settings. Moreover, although the 32‐day incubation provided insights into early nutrient responses, the short incubation duration limits understanding of long‐term persistence and trajectory of the observed effects. Furthermore, our comparison was based on equal application rates by mass and captures primarily the nutrient density effects of biochar, reflecting typical agronomic practices where amendments are applied at similar area‐based rates. In practice, pyrolyzing straw substantially reduces its mass, so the amount of biochar produced from a given quantity of straw would be lower.

Future studies should evaluate a broader range of soils, biochar feedstocks, pyrolysis conditions, application strategies, and longer term nutrient dynamics to validate the consistency of these effects and enhance the general applicability of these observations. Since biochar may enhance nutrient availability either through dissolution of its inherent nutrients or by mobilizing soil‐bound pools, future studies should employ tracking techniques such as stable isotope labeling to distinguish these mechanisms and quantify their relative contributions. Future studies could also consider comparisons based on actual conversion yields, which would more closely reflect the practical implications of straw‐to‐biochar conversion.

## 5 CONCLUSION

The conversion of wheat straw to biochar may contribute a dual solution to both efficient disposal of waste and reutilization as soil amendment, addressing a critical research gap by generating new evidence for fertile Luvisols—a soil type that has received less attention compared to acidic soils. Biochar produced at higher pyrolysis temperatures (500°C at 2 h residence time and 650°C at both 1 and 2 h residence times) proved most effective at enhancing soil available nutrients, particularly P and K. Moreover, the amounts of soil available P and K in the Luvisols soil benefited from both practical application rates, with the enhancement being much greater at the higher practical rate (10 Mg ha^−1^) than at the lower rate (5 Mg ha^−1^). This observed increase in available nutrients did not significantly differ whether the biochar was incorporated into the soil or applied as surface broadcast, suggesting that conventional and no‐tillage systems could equally benefit from using biochar for P and K supplementations. In contrast, the effects on N were inconsistent, with no significant improvement observed and some treatments indicating possible immobilization. We acknowledge that the outcomes of the current study originate from our specific scenario in a controlled laboratory microcosm experiment, which limits direct applicability to field conditions, especially in the use of a high biochar rate (20 Mg ha^−1^). Future studies, therefore, should investigate how various pyrolysis conditions and application strategies of biochar influence long‐term nutrient cycling and crop responses under variable field conditions and across diverse Luvisols. Such research will refine recommendations tailored to soil characteristics, pyrolysis conditions, application rates, and farming practices.

## AUTHOR CONTRIBUTIONS

**Syazwan Sulaiman**: Conceptualization; data curation; formal analysis; funding acquisition; investigation; methodology; visualization; writing—original draft; writing—review and editing. **Guillermo Hernandez‐Ramirez**: Funding acquisition; resources; supervision; writing—review and editing. **Namasivayam Navaranjan**: Supervision. **Zohrah Sulaiman**: Supervision.

## CONFLICT OF INTEREST STATEMENT

The authors declare no conflicts of interest.

## Data Availability

The data that support the findings of this study are available from the corresponding author upon reasonable request.
